# Phacomatosis pigmentokeratotica and precocious puberty associated with *HRAS* mutation

**DOI:** 10.1111/bjd.15643

**Published:** 2017-11-27

**Authors:** R.J. Martin, M. Arefi, M. Splitt, L. Redford, C. Moss, N. Rajan

**Affiliations:** ^1^ Department of Clinical Genetics Centre for Life Newcastle upon Tyne U.K.; ^2^ Institute of Genetic Medicine Newcastle University Newcastle upon Tyne U.K.; ^3^ Department of Dermatology Birmingham Children's Hospital University of Birmingham Birmingham U.K.

dear editor, Germline mutations in the oncogene *HRAS* cause syndromes with systemic and cutaneous features, notably Costello syndrome (CS).^1^ Postzygotic activating mutations in *HRAS* are increasingly recognized as a cause of epidermal naevi that are sometimes associated with the extracutaneous features of germline rasopathies. Epidermal naevi caused by *HRAS* mutations present with varied morphology including sebaceous naevus, woolly hair naevus and phacomatosis pigmentokeratotica (PPK).^2^

PPK is characterized by the association of epidermal naevus with speckled lentiginous naevus of the papular type or papular naevus spilus. To some extent these localized epidermal phenotypes correspond to the more generalized cutaneous features of CS, namely acanthosis nigricans, papillomas and curly hair. Pigmented patches and increased numbers of melanocytic naevi also occur in CS^3^ but are more characteristic of another RASopathy, cardiofaciocutaneous syndrome, caused by *BRAF* mutations.

We report an informative patient with PPK, localized curly hair, precocious puberty and a mosaic activating *HRAS* mutation. This white male patient first presented aged 2 years^4^ with enlarged genitalia, pubic hair, accelerated growth, extensive epidermal naevi and multiple melanocytic naevi. Endocrine investigations confirmed central precocious puberty with adult levels of luteinizing hormone and testosterone. Computerized tomography and contrast magnetic resonance imaging of his brain at age 12 years showed no pituitary tumour or any other intracranial anomaly. His medical and family history were otherwise noncontributory.

Re‐examination at age 33 years revealed an extensive Blaschkoid epidermal naevus affecting the face, neck and upper torso (Fig. 1a–d). Some keratotic areas on the posterior neck, in keeping with a sebaceous naevus, had been laser ablated. A large papular naevus spilus on the left anterior torso partly overlapped the epidermal naevus. On the forearms, papular naevus spilus and epidermal naevi colocalized, while on the right calf there was papular naevus spilus alone. The scalp hair was brown with a striking Blaschkoid pattern of lighter, shorter, wavy hair colocalizing with melanocytic naevi but no apparent alopecia as typically seen in sebaceous naevus involving the scalp.

**Figure 1:**
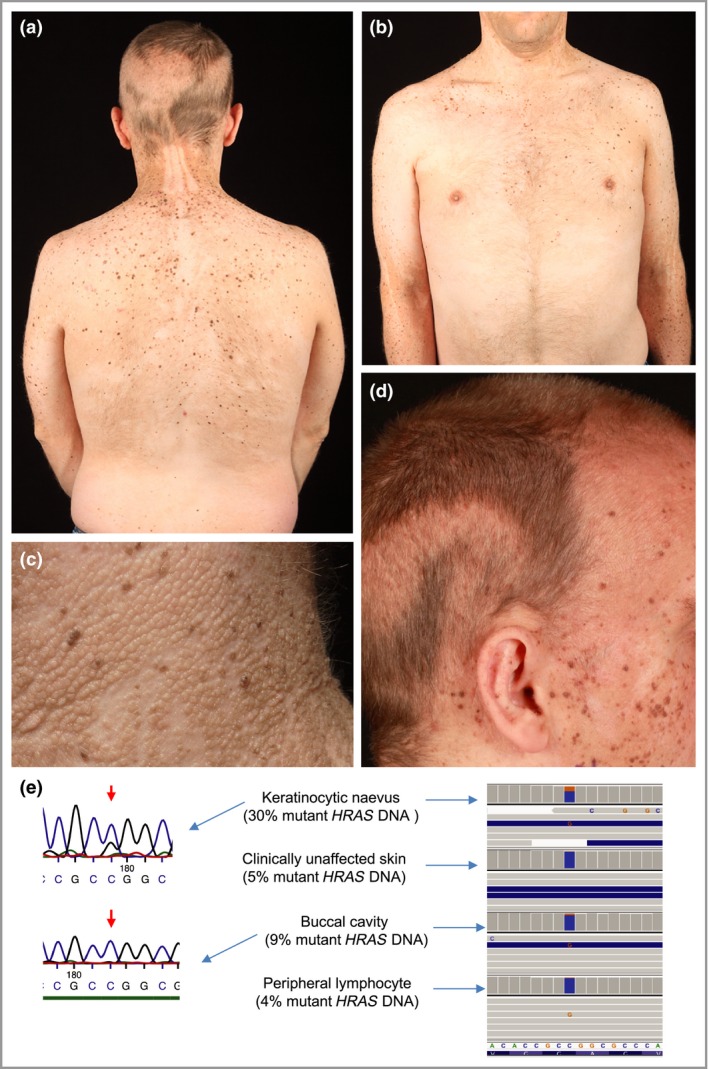
Clinical images of the patient aged 33 years and genetic analysis. (a,b) Head and torso demonstrating linear and patchy lesions of sebaceous naevus and patchy lesions of papular naevus spilus, partly overlapping each other. (c) Close‐up of the neck revealing the sebaceous naevus at this site of the epidermal naevus. (d) Blaschkoid whorls are seen within the scalp with lighter, wavy hair. (e) DNA from the epidermal naevus but not buccal mucosa appears to carry the *HRAS* c.34G>C variant demonstrated by Sanger sequencing; targeted next‐generation sequencing reveals mutant *HRAS* reads (visualized in Integrative Genomics Viewer^7^) in the epidermal naevus, as well as in unaffected skin, saliva and blood.

Following informed consent and ethics committee approval, skin samples were taken from the neck where epidermal naevus and papular naevus spilus overlapped and unaffected skin from the lower back at the patient's request, together with a buccal swab and blood sample. DNA was extracted and targeted deep sequencing performed for hotspots in *BRAF, HRAS, KRAS* and *NRAS*. A rarely reported, activating mutation at codon 12 of *HRAS* (c.34G>C; p.Gly12Arg) was detected in affected skin (30% mutant *HRAS* reads) and unaffected skin (5% mutant *HRAS* reads) (mean coverage of 29000X); no other changes were detected in the remaining targeted regions (Fig. 1e). DNA from buccal mucosa did not display the mutation using Sanger sequencing but deep sequencing revealed a low percentage of mutant *HRAS* in saliva (9%) and blood (4%).

Extracutaneous features in PPK typically affect neurological, skeletal and endocrine systems. Hypophosphataemic rickets due to cutaneous skeletal hypophosphataemia syndrome is sometimes associated with verrucous epidermal naevi particularly in the context of PPK and is attributed to fibroblast growth factor (FGF)23 production by bone lesions not detected in our patient. Precocious puberty, seen rarely in CS,^5^ is a recurrent finding in patients with PPK, occurring in eight of 30 reported cases including ours (Table S1; see Supporting Information). The only previous case of this association where mutation analysis was performed showed a somatic *BRAF* mutation (p.Lys601Asn).^6^

The *HRAS* mutation reported here was found not only in affected skin but also in clinically normal tissues, a feature not demonstrated in the four previous cases of PPK with extracutaneous features attributed to mosaic RASopathy.^2^, ^6^ This disparity may reflect the greater sensitivity of the targeted sequencing strategy we used and suggests that *HRAS* mutant cells may occur in other, inaccessible organs such as the pituitary gland. Given the association noted above of precocious puberty with PPK and by implication with mosaic RASopathy, a causal relationship seems likely. We suggest that pituitary *HRAS* mosaicism disturbs cellular homeostasis in some way, perhaps involving a second messenger analogous to HRAS‐induced FGF23, leading to central precocious puberty, but the precise mechanism remains obscure. Taken together with the report by Kuentz *et al*.,^6^ these cases strongly implicate Ras‐Raf‐MEK‐ERK signalling in the development of central precocious puberty, with important therapeutic potential.

In conclusion, this unusual case demonstrates parallels between PPK and CS reflecting, respectively, mosaic and germline HRASopathy. We showed mutant *HRAS* DNA in the unaffected buccal mucosa and blood of a patient with PPK with extracutaneous features. We confirmed an association of PPK with precocious puberty. Finally, our observation offers insight into the effects of Ras‐Raf‐MEK‐ERK pathway activation on pituitary function as well as the differential responses of epidermal cell subpopulations.

## Supporting information


**Table S1** Reported patients with phacomatosis pigmentokeratotica and precocious puberty.
