# Supplementary material for: Phacomatosis pigmentokeratotica and precocious puberty associated with HRAS mutation
Source: Br J Dermatol. 2017 Nov 27;178(1):289–91. doi: 10.1111/bjd.15643 (PMC5961630; doi:10.1111/bjd.15643)
Supplement: Supplementary file 1 — Table S1 Reported patients with phacomatosis pigmentokeratotica and precocious puberty. [file BJD-178-289-s001.docx]

**Phacomatosis pigmentokeratotica and precocious puberty associated with *HRAS* mutation.**

RJ Martin^1^, M Arefi^2^, M Splitt^1^, L Redford^2^, C Moss^3^, N Rajan^2^.

Supplementary File containing Table S1

**Table S1** Reported patients with phacomatosis pigmentokeratotica (PPK) and precocious puberty

| **Case** | **Genetic analysis** | **Skin phenotype*** | **Onset of precocious puberty** | **Other features** | **Reference** |  |
| --- | --- | --- | --- | --- | --- | --- |
| 1 | HRAS c.34G>C; p.Gly12Arg | Linear epidermal naevus | 18 months | Urinary tract anomalies, hypercholesterolaemia | Moss^1^ |  |
| 2 | BRAF p.Lys601Asn | PPK with woolly hair naevus | Not indicated | Focal cortical dysplasia, epilepsy, learning difficulties | Kuentz^2^ |  |
| 3 | Not tested | Systematized verrucous epidermal naevus | 3 years | Developmental delay, epilepsy | Garg^3^ |  |
| 4 | Not tested | Epidermal naevus | 14 months | Congenital rhabdomyosarcoma, hemihypertrophy, hypophosphataemic rickets | Shahgholi^4^ |  |
| 5 | Not tested | Linear naevus sebaceous (Schimmelpenning–Feuerstein–Mims) syndrome | 9 years | Ocular abnormalities, hypophosphataemic rickets | Zutt^5^ |  |
| 6 | Not tested | Linear epidermal naevus | 11 months | Hypophosphataemic rickets, congenital partial 3^rd^ nerve palsy | Ivker^6^ |  |
| 7 | Not tested | Epidermal naevus and woolly hair naevus | 20 months | None | Tay^7^ |  |
| 8 | Not tested | Epidermal naevus | 9 years | Polyostotic fibrous dysplasia, intracranial lipomas, unilateral ventriculomegaly, conjunctival dermis and coloboma | Yu^8^ |  |

*The skin phenotype reported here is based on the respective author’s reports. In several cases it should be noted that papular speckled naevus may be seen in the supplied photographs, supporting a diagnosis of PPK.

**Supplementary references**

1 Moss C, Parkin JM, Comaish JS. Precocious puberty in a boy with a widespread linear epidermal naevus. *Br J Dermatol* 1991; **125**:178–82.

2 Kuentz P, Mignot C, St-Onge J *et al.* Postzygotic BRAF p.Lys601Asn mutation in phacomatosis pigmentokeratotica with woolly hair nevus and focal cortical dysplasia. *J Invest Dermatol* 2016; **136**:1060–2.

3 Garg T, Chander R, Gaur N *et al.* Precocious puberty in a 3-year-old child with systematized verrucous epidermal nevus. *Indian J Dermatol Venereol Leprol* 2015; **81**:197–8.

4 Shahgholi E, Mollaian M, Haghshenas Z *et al.* Congenital rhabdomyosarcoma, central precocious puberty, hemihypertrophy and hypophosphatemic rickets associated with epidermal nevus syndrome. *J Pediatr Endocrinol Metab* 2011; **24**:1063–6.

5 Zutt M, Strutz F, Happle R *et al.* Schimmelpenning-Feuerstein-Mims syndrome with hypophosphatemic rickets. *Dermatology* 2003; **207**:72–6.

6 Ivker R, Resnick SD, Skidmore RA. Hypophosphatemic vitamin D-resistant rickets, precocious puberty, and the epidermal nevus syndrome. *Arch Dermatol* 1997; **133**:1557–61.

7 Tay YK, Weston WL, Ganong CA *et al.* Epidermal nevus syndrome: association with central precocious puberty and woolly hair nevus. *J Am Acad Dermatol* 1996; **35**:839–42.

8 Yu AC, Ng V, Dicks-Mireaux C *et al.* Epidermal naevus syndrome associated with polyostotic fibrous dysplasia and central precocious puberty. *Eur J Pediatr* 1995; **154**:102–4.
